# Keystone Veterinary Medical Association

**Published:** 1895-06

**Authors:** W. L. Rhoads

**Affiliations:** Secretary


### SOCIETY PROCEEDINGS.

### KEYSTONE VETERINARY MEDICAL ASSOCIATION.

THE monthly meeting was held at the office of Dr. W. H. Hoskins on the 21st ult., having been postponed from the 14th, the regular night of meeting.

The meeting was called to order by President Lintz. Then followed roll-call, reading and adoption of minutes of previous meeting, and committee reports, during which it was learned that the bill to establish a State Board of Veterinary Medical Examiners needs but the Governor's signature to become a law.

On motion, President Lintz appointed Drs. Hart and Rhoads as a committee of two to confer with the State Association as to what could be done toward having men appointed on this board who were willing to make personal sacrifices for the advancement of the profession, and not men who want it solely for self-aggrandizement and personal advancement it would bring them, and whose only claim or hope of obtaining it is the political influence they they can bring to bear.

The meeting adjourned to meet at Lansdowne, June 11th, where they will they will be entertained by Dr. Rhoads. W. L. RHOADS,

Secretary.

